# Human neural stem cell-derived extracellular vesicles mitigate hallmarks of Alzheimer’s disease

**DOI:** 10.1186/s13195-021-00791-x

**Published:** 2021-03-06

**Authors:** Lauren A. Apodaca, Al Anoud D. Baddour, Camilo Garcia, Leila Alikhani, Erich Giedzinski, Ning Ru, Anshu Agrawal, Munjal M. Acharya, Janet E. Baulch

**Affiliations:** 1 Department of Radiation Oncology, University of California Irvine, Irvine, CA 92697 USA; 2 Department of Medicine, University of California Irvine, Irvine, CA 92697 USA

**Keywords:** Alzheimer’s disease, Extracellular vesicle, Neural stem cell, Inflammatory response

## Abstract

**Background:**

Regenerative therapies to mitigate Alzheimer’s disease (AD) neuropathology have shown very limited success. In the recent era, extracellular vesicles (EVs) derived from multipotent and pluripotent stem cells have shown considerable promise for the treatment of dementia and many neurodegenerative conditions.

**Methods:**

Using the 5xFAD accelerated transgenic mouse model of AD, we now show the regenerative potential of human neural stem cell (hNSC)-derived EVs on the neurocognitive and neuropathologic hallmarks in the AD brain. Two- or 6-month-old 5xFAD mice received single or two intra-venous (retro-orbital vein, RO) injections of hNSC-derived EVs, respectively.

**Results:**

RO treatment using hNSC-derived EVs restored fear extinction memory consolidation and reduced anxiety-related behaviors 4–6 weeks post-injection. EV treatment also significantly reduced dense core amyloid-beta plaque accumulation and microglial activation in both age groups. These results correlated with partial restoration of homeostatic levels of circulating pro-inflammatory cytokines in the AD mice. Importantly, EV treatment protected against synaptic loss in the AD brain that paralleled improved cognition. MiRNA analysis of the EV cargo revealed promising candidates targeting neuroinflammation and synaptic function.

**Conclusions:**

Collectively, these data demonstrate the neuroprotective effects of systemic administration of stem cell-derived EVs for remediation of behavioral and molecular AD neuropathologies.

**Supplementary Information:**

The online version contains supplementary material available at 10.1186/s13195-021-00791-x.

## Background

The most common form of dementia is Alzheimer’s disease (AD), an irreversible neurodegenerative disease that is characterized by progressive cognitive and functional decline and memory loss [1]. Currently, there are no effective therapies to slow or reverse the disease progression. Hallmarks of AD include amyloid-beta (Aβ) plaques, neurofibrillary tangles, and neuroinflammation which is accompanied by dementia. Microglia, the resident macrophages of the brain, become activated to engulf and clear the extracellular Aβ plaque deposits. However, persistent microglial activation in AD eventually leads to a pro-inflammatory environment that includes increased expression of pro-inflammatory cytokines and reduced expression of anti-inflammatory cytokines [2–4]. Ultimately, this chronic inflammation, in addition to the plaque deposition, creates a neurotoxic microenvironment that leads to loss of dendritic spines and neurons in the AD brain [4].

Similar neuropathologies including persistent inflammation, loss of synaptic integrity, and cognitive impairments have been clearly shown to manifest in the radiation-damaged brain [5]. In a series of studies, we demonstrated that hippocampal engraftment of human neural stem cell (hNSC) ameliorated neurocognitive and neuroinflammatory effects of a clinically relevant radiation exposure using athymic nude rats [6–9]. These studies showed that stem cell-based approaches improved the functional plasticity of the irradiated host brain, suppressing neuroinflammation, preserving host neuronal morphology, and improving cognitive function. Since significant attrition of engrafted cells was observed, it was hypothesized that the hNSC provided neuroprotective benefits primarily via trophic support rather than proliferation and repopulation [10–12].

A somewhat similar trajectory involving stem cells has been observed in the search for effective approaches to ameliorate AD neuropathology. Studies have demonstrated that hNSC transplantation can provide at least some neurocognitive benefits in transgenic mouse models of AD [13]. The data from both radiation and AD studies have suggested that the neuroprotective properties and trophic support mechanisms by which NSC act to reduce inflammation and preserve the structural integrity of the injured brain are via the stem cell secretome, more specifically stem cell-derived extracellular vesicles (EVs) [10–12, 14, 15]. In the case of cranial irradiation, this assertion has been borne out through a series of studies, using hNSC-derived EVs. In a first study, the efficacy of EV therapy was demonstrated in an irradiated athymic nude rat model [10, 12] and then in a study using immunocompetent mice [11]. Studies by others have similarly indicated that mouse stem cell-derived EV transplantation can provide neurocognitive benefits in transgenic mouse models of AD [14, 15].

EVs are small membrane-bound vesicles that are less immunoreactive than their cellular source and contain bioactive cargo such as proteins, mRNA, and microRNA (miRNA) [16]. Endogenously, EVs are recognized as important modulators of physiological processes, with specific cargo capable of controlling cell signaling and target cell function to maintain homeostasis or to contribute to disease pathology [17–19]. Exogenous stem cell-derived EVs represent a unique paracrine signaling mechanism with great therapeutic potential, particularly in the context of CNS injuries such as traumatic brain injury, stroke, and neurodegenerative diseases [14, 15, 19–22]. These EVs not only cross the blood-brain barrier but also negate concerns regarding the possibility of teratoma formation and immune rejection that confound related stem cell-based approaches [23–25]. Immune rejection is a particularly significant problem in the case of AD, since the long-term use of immunosuppressant can result in toxicity, particularly in the aged, and may also exacerbate AD-related pathologies [22, 26].

In this study, the 5xFAD mouse model of AD that co-expresses five human familial AD mutations was used [27]. The 5xFAD mouse is an accelerated model of AD with measurable Aβ plaque load by 2 months of age but in the absence of neurofibrillary tangles. These animals also exhibit reductions in synaptophysin, neuronal loss, and memory impairments that become evident by 5–6 months of age. The goal of this study was to test the hypothesis that hNSC-derived EV treatment could mitigate AD-related behavioral and molecular neuropathologies when delivered intravenously to early- or late-stage AD mice.

## Materials and methods

### hNSC culture and EV isolation and characterization

The use of hNSC was approved by the Institutional Human Stem Cell Research Oversight Committee (HSCRO). The validation, expansion, and characterization of hNSC (ENStem-A; EMD Millipore) followed previously published procedures [28]. Before isolation of extracellular vesicles (EVs), the conditioned hNSC culture medium was put through a 0.22-μm sterile filter as an initial purification step before centrifugation. EVs were then isolated and further purified from the hNSC culture medium by ultracentrifugation [10, 29] and characterized for size and number by a ZetaView particle analyzer as per instrument instructions (ZetaView PMX 110; Meerbusch, Germany).

EVs were also analyzed by electron microscopy for morphology where the material was negatively stained by applying a drop of EV solution (final concentration approximately 1 mg/ml) directly onto a 300-mesh formvar-carbon-coated nickel grid (Electron Microscopy Sciences; Hatfield, PA), which was allowed to remain for approximately 60 s, after which excess solution was removed. A drop of 1% aqueous uranyl acetate was then added onto the grid and allowed to remain for an additional 60 s, after which excess solution was removed and the grids allowed to dry. Material was imaged on a Hitachi 7500 transmission electron microscope equipped with an Advanced Imaging Technologies (AMT) digital camera. A digital point-to-point measuring tool (AMT) was used to determine exosome size distribution. Images were imported into Photoshop CS2 (Adobe Systems Inc.) where they were sized and optimized for contrast and brightness.

### Animals and EV delivery

All animal experimentation procedures were performed under the guidelines provided by NIH and approved by the University of California Irvine Institutional Animal Care and Use Committee. Male 5xFAD transgenic mice and age-matched littermate controls were maintained in standard housing conditions (20 °C ± 1 °C; 70% ± 10% humidity; 12-h:12-h light and dark cycle) and provided ad libitum access to standard rodent chow (Envigo Teklad 2020x) and water. For the early-stage AD study, male 5xFAD mice and their wild type littermate controls were divided into the following groups: vehicle-injected AD with sterile hibernation buffer (Gibco) (AD; *N* = 15), EV-injected AD (AD+EV; *N* = 16), and vehicle-injected wild type (WT; *N* = 16). The mice were 1.5–2.5 months of age at the time of EV treatment and stratified by age to maintain equivalent age distributions among experimental groups. Behavior studies were initiated 1-month post-EV treatment when mice were 2.5–3.5 months of age. The late AD study utilized the same treatments and stratification of male mice where the AD mice and their WT littermate controls were 5.0–6.5 months of age (*N* = 14–16 AD, AD+EV, WT) at the first EV treatment. A second EV injection was administered 2 weeks later. One month after the second EV treatment, when the mice reached 6.5–8.0 months of age, behavior studies were initiated.

Our previous published studies demonstrated equal effectiveness of EV treatment using both intra-hippocampal stereotaxic surgery and injection into the retro-orbital sinus (RO) [11]. Therefore, the relatively non-invasive RO injection route of EV administration was used in this study. To administer EVs, mice were sedated using 2.5% (v/v) isoflurane/oxygen, and 2.25 × 10^7^ EVs in 50 μl hibernation buffer were delivered into circulation via RO injection. Control AD and WT mice received RO injections on that same schedule using 50 μl of vehicle. Over the years, our group, and others, have not included grafted controls (either stem cells or EVs) since such transplantation procedures used to treat a variety of pathologies in different rodent models were not found to functionally affect the intact normal brain [10, 30–33]. Importantly, our past work using stem cells [34] and from others using EVs [35, 36], in which grafted controls were included, found that all functional and molecular endpoints were statistically indistinguishable between the control, stem cell-treated control, or EV-treated control groups. Further rationale for excluding controls treated with EVs alone is that inclusion of such cohorts is clinically irrelevant.

### Behavioral testing

Behavioral studies began 1 month after EV treatment using *N* = 15–16 mice per group as described above. Testing occurred over 3 weeks and included the following paradigms in order: novel object recognition (NOR), elevated plus maze (EPM), light-dark box (LDB), and fear extinction (FE) memory tasks. Independent investigators blinded to the experimental groups scored all behavior videos. NOR, EPM, and LDB were scored manually, while FE was scored for freezing using FreezeFrame (Coulbourn Instruments). See Supplemental Information for details.

The spontaneous exploration task NOR relies on intact hippocampal, medial prefrontal cortex (mPFC), and perirhinal cortex (PRC) function, evaluating episodic recognition memory by measuring the preference of mice to investigate novelty [37, 38]. Mice were habituated to the arena with only bedding for 3 days followed by a test day where mice were presented with two plastic objects that were similar in color, shape, and size. Each mouse was returned to the home cage for 5 min while one familiar object was substituted for a novel object. The mouse was then returned to the arena for 5 min of further exploration. The discrimination index, as measured by the time spent interacting with familiar versus novel objects, was then calculated for each mouse from these values: [(novel/total exploration time) – (familiar/total exploration time)] × 100.

The EPM and LDB tests are based on the tendency of anxious rodents to avoid open or brightly lit areas and to exhibit reluctance to explore open environments, resulting in reduced amounts of time spent in the open arms of the EPM or reduced numbers of transitions between the dark and light compartments of the LDB testing arena [39]. The EPM consists of 2 open arms and 2 closed arms arranged in the shape of a plus sign [40, 41]. Each mouse was placed in the neutral center zone of the plus maze and allowed to explore for 5 min. Anxiety-like behavior was scored as the percent time spent in the open arms of the maze as compared to the closed arms. Following EPM, the next day, mice were tested on LDB where anxiety was measured by a mouse’s willingness to transition freely between a large well-lit chamber to a smaller, dimly lit chamber. Fewer transitions and less time spent in the well-lit chamber during the 10-min test suggested increased anxiety-like behavior [39].

The last behavior test administered was the FE test where two contexts (A and B) were used to determine whether mice could learn and then extinguish conditioned fear responses over 5 days [42, 43]. The conditioning testing chamber (context A) had a steel grid floor and the scent of acetic acid, while the extinction chamber (context B) had a smooth Plexiglas floor, additional stimulus lighting, and the scent of almond extract. Freezing behavior was recorded via digital cameras mounted in the ceiling of each chamber automated scoring (FreezeFrame, Coulbourn Instruments). For each mouse, the fear conditioning protocol for day 1 used context A and started with a 120-s habituation phase followed by 3 pairings of a 120-s white noise conditioned stimulus (CS) co-terminating with a 1-s foot shock (US) presented at 2-min intervals (day 1, *T*_1_–*T*_3_). For extinction training, starting on day 2, each mouse was placed in context B and allowed to acclimate for 2 min followed by extinction training that was comprised of 15 non-reinforced 120-s CS presentations presented graphically as the average of 5 tones. Extinction training was repeated daily for 2 additional days. Subsequently, retention testing was performed on day 5 where each mouse was returned to context B and following a 2-min acclimation period freezing was assessed during three non-US-reinforced CS tones at 2-min intervals. Extinction memory was calculated as the percentage of time spent freezing during the test.

### Thioflavin-S (Thio-S) staining, immunohistochemistry, and confocal microscopy analysis

Following behavior, a subset of those same mice was deeply anesthetized using isoflurane and euthanized via intracardiac perfusion using 4% paraformaldehyde (Sigma) in 100 mM phosphate-buffered saline (PBS; pH 7.4, ThermoFisher Scientific). Brains were cryoprotected (10–30% sucrose gradient over 2–3 days) and sectioned coronally into 30 μm using a cryostat (Leica Microsystems, Germany). For each endpoint, 4 representative coronal brain sections of the amygdala and medial prefrontal cortex (mPFC) regions from each of the 4 animals per experimental group were selected at approximately 15 section intervals, washed in PBS, and stored in PBS. Sections were rehydrated in ethanol gradients (100%, 95%, 70%, 50%) and then incubated in a 0.5% Thio-S solution in 50% ethanol for 10 min. Tissues were rinsed twice in 50% ethanol and then rinsed twice in PBS. Sections were mounted and sealed with slow fade/gold antifade mounting medium (Life Technologies). Confocal imaging was conducted using Nikon Eclipse Ti C2 microscope (Nikon, Japan) equipped with a 20× lens and NIS-Elements AR interface (v4.30, Nikon). Approximately, 20–25 z stacks (1 μm thick) were collected that were uploaded to AutoQuant deconvolution module (version X3.0.4, Media Cybernetics, Rockville, MD) followed by direct recall into the Imaris module (version 8.1.2, Bitplane, Inc., Zurich, Switzerland) to create surface reconstruction of the deconvoluted, Thio-S-positive (green) surfaces. The number of Thio-S-positive plaques per section was reported to determine the extent of plaque deposition in each group.

Immunohistochemistry was also performed on the PFA-fixed brain sections. For the immunofluorescence labeling of microglial activation marker CD68 and pre-synaptic marker synaptophysin, rat anti-mouse CD68 (1:500; AbD Serotec) and mouse-anti-synaptophysin (1:1000, Sigma) primary antibodies, respectively, were used with Alexa Fluor 568 secondary antibody (1:500 and 1:1000, respectively). Tissues were then DAPI nuclear counterstained and sealed in slow fade/gold antifade mounting medium (Life Technologies).

The stained coronal brain sections were scanned using a confocal microscope (Nikon Eclipse Ti C2) equipped with a 40× PlanApo oil-immersion lens (1.3 NA, Nikon) and an NIS-Elements AR interface (v4.30, Nikon). Thirty z stacks (1024 bit depth) at 0.5 μm from three different fields (318 × 318 × 24 μm) were imaged in each section in the areas of interest. The digitized z stacks were deconvoluted using the AutoQuant software. An adaptive, 3D blinded method was used to create deconvoluted images for direct import into the Imaris module. The 3D algorithm-based surface rendering and quantification of fluorescence intensity for each fluorescently labeled marker was carried out in Imaris at 100% rendering quality. Each channel was analyzed separately. 3D surface rendering detects immunostained puncta or nuclear staining (DAPI) satisfying pre-defined criteria, for the puncta size (0.5 to 1 μm) verified visually for accuracy. Using deconvoluted confocal z stack volume from the control group (WT) as a baseline for the minimum thresholding, a channel mean intensity filter was applied and used for all the experimental groups for each batch of molecular markers. The pre-set parameters were kept constant throughout the subsequent analysis of immunoreactivity for each antigen. To maintain uniformity among the varying number of puncta for each time point and/or antigen analyzed, the number of puncta per 318 × 318 × 24 μm was normalized to WT control, and data was expressed as percent mean immunoreactivity relative to WT controls.

### Amyloid-β ELISA

Fresh frozen brain tissues were isolated from another subset of the behaviorally tested mice. As per the manufacture’s protocol, isolated protein samples from fresh frozen brains were applied to a blocked MSD Human/Rodent (4G8) Aβ triplex ELISA plate and incubated for 2 h at room temperature on an orbital shaker (Aβ_1–38_, Aβ_1–40_, Aβ_1–42_; Meso Scale Discovery, Rockville MD). The plate was then washed, and measurements obtained using a SECTOR Imager 2400. *N* = 7 and 6 mice per experimental group for AD and AD+EV soluble Aβ measurements and *N* = 7 mice per experimental group for insoluble Aβ measurements, with 1 WT negative control for both measurements.

### Spleen cytokine analysis and flow cytometry

Fresh spleens were collected from early- and late-stage AD, AD+EV, and WT mice, 7 mice per group. For cytokine analysis, spleen cells (1 × 0^6^/ml) were stimulated with phorbol 12-myristate 13-acetate (PMA; 50 ng/ml; Sigma) and ionomycin (1 μg/ml; Sigma) in RPMI medium containing 10% FBS. Supernatants collected after overnight stimulation were assayed for IFN-γ, IL-17, and IL-10, IL-1β, using a magnetic bead-based kit (Thermo Fisher Scientific). Single-cell spleen suspensions were stained with antibodies specific to B1 cells (CD19^+^CD5^+^CD43^+^; Biolegend, San Diego, CA). The analysis was done using Flow Jo software (Treestar, Ashland, OR).

### Gene expression analysis

Total mRNA was purified from the fresh frozen hippocampus of each of 4 mice per group, WT, AD, and AD+EV, from the late-stage AD cohort and multiplexed using the nCounter Mouse Neuroinflammation Panel that includes 757 genes that cover core pathways that define neuroinflammation processes and 13 internal reference genes (NanoString, CA).

Counts for target genes were normalized to the housekeeping genes to account for variability in RNA content. Background signal was calculated as a mean value of the negative hybridization control probes. Gene expression values were presented as the percentage of the WT control group for comparison of the AD cohort to the AD+EV cohort.

### MicroRNA microarray and validation

The hNSC-derived EVs were lysed using Qiazol and miRNA isolated using the Qiagen miRNeasy kit as per the manufacturer’s protocol (Qiagen, CA). Samples were analyzed for integrity and concentration (NanoDrop; 260/280 ratio > 1.6 and 260/230 ratio > 1.5), then processed and analyzed in duplicate on a miRNA microarray chip (Exiqon, Denmark; Genomics Shared Resource at the University of Texas South Western Medical Center). Results were delivered as a spreadsheet of miRNA IDs and their associated expression values. Negative control probes were included for the determination of significant hits. Another spreadsheet provided was filtered for probes that had duplicate measurements with less than 15% coefficient of variation (CV) and at least three standard deviations greater than the negative control probes.

Select candidate miRNA from the array were identified through literature searches of miRNA implicated in AD pathologies. Validation of those candidate miRNAs was performed using TaqMan Advanced miRNA Assays (ThermoFisher, MA). Total RNA was extracted from two independent EV preparations using the RNA miniprep kit (Zymo Research Corp., CA). RNA template was then ligated to adaptors and pre-amplified using the TaqMan Advanced miRNA cDNA Synthesis Kit (ThermoFisher) as per the manufacturer’s protocol to obtain the cDNA template for classical qPCR using specific TaqMan Advanced miRNA Assays (ThermoFisher) which are primers specific to the target miRNA. Duplicate reactions were set up in a 96-well plate with no-template controls (MilliQ water instead of total RNA was used for cDNA synthesis process) included for each assay. Cycling was performed in the CFX96 (BioRad Laboratories, Inc., CA). qPCR data were visualized and processed using CFX Manager software (BioRad Laboratories, Inc., CA).

### Statistical analysis

Statistical analyses were carried out using GraphPad Prism (v6). The Shapiro-Wilk test was used to assess the normal distribution of all behavioral testing data. Unless stated otherwise, one-way analysis of variance (ANOVA) was used to assess the significance for the AD, AD+EV, and WT groups of mice. When overall group effects were found to be statistically significant, Bonferroni’s multiple comparisons test was used to compare the AD group with each of the other experimental groups. A *P* value of ≤ 0.05 was considered to be statistically significant.

## Results

### EV characterization and experimental design

**Fig. 1:**
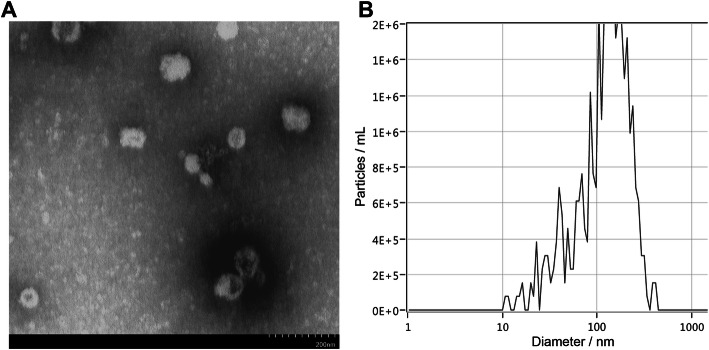
Characterization of extracellular vesicles. **a** Representative electron microscopy image depicts typical EV morphology and size. **b** Representative graph from ZetaView analysis showing the average size distribution of the EVs

**Fig. 2:**
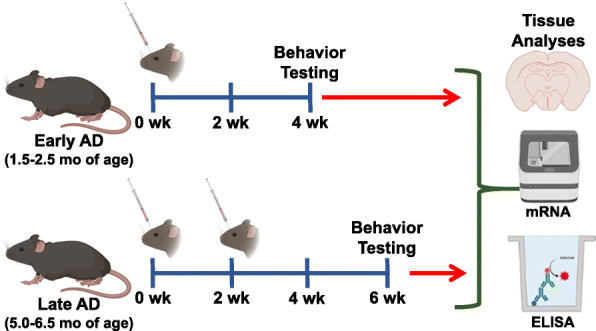
Experimental design. Early AD male mice were treated with a single dose of human neural stem cell-derived extracellular vesicles (EVs) via retro-orbital vein (RO) injection at ~ 1.5–2.5 months of age and behaviorally tested 1 month later. Late-stage AD male mice received 2 RO injections of EVs at 2-week intervals and began behavior testing 1 month after the second injection

EVs were characterized for morphology by electron microscopy and for size and number using a ZetaView particle analyzer. The mean EV size was 147.8 ± 2.1 nm diameter at a mean stock concentration of 7.58 × 10^9^ ± 6.40 × 10^8^ EV per ml (Fig. 1). The purified EVs were diluted into sterile hibernation buffer to deliver 2.25 × 10^7^ EV per 50 μl RO injection. For the early-stage AD study, male 5xFAD mice and their wild type littermate controls were 1.5–2.5 months of age at the time of EV treatment and behavior studies were initiated 1-month post-EV treatment when mice were 2.5–3.5 months of age. The late AD study utilized the same treatments and stratification of male mice where the AD mice and their WT littermate controls were 5.0–6.5 months of age at the first EV treatment. A second EV injection was administered 2 weeks later. One month after the second EV treatment, when the mice reached 6.5–8.0 months of age, behavior studies were initiated (Fig. 2).

### Effects of disease progression and EV treatment on cognitive function

**Fig. 3:**
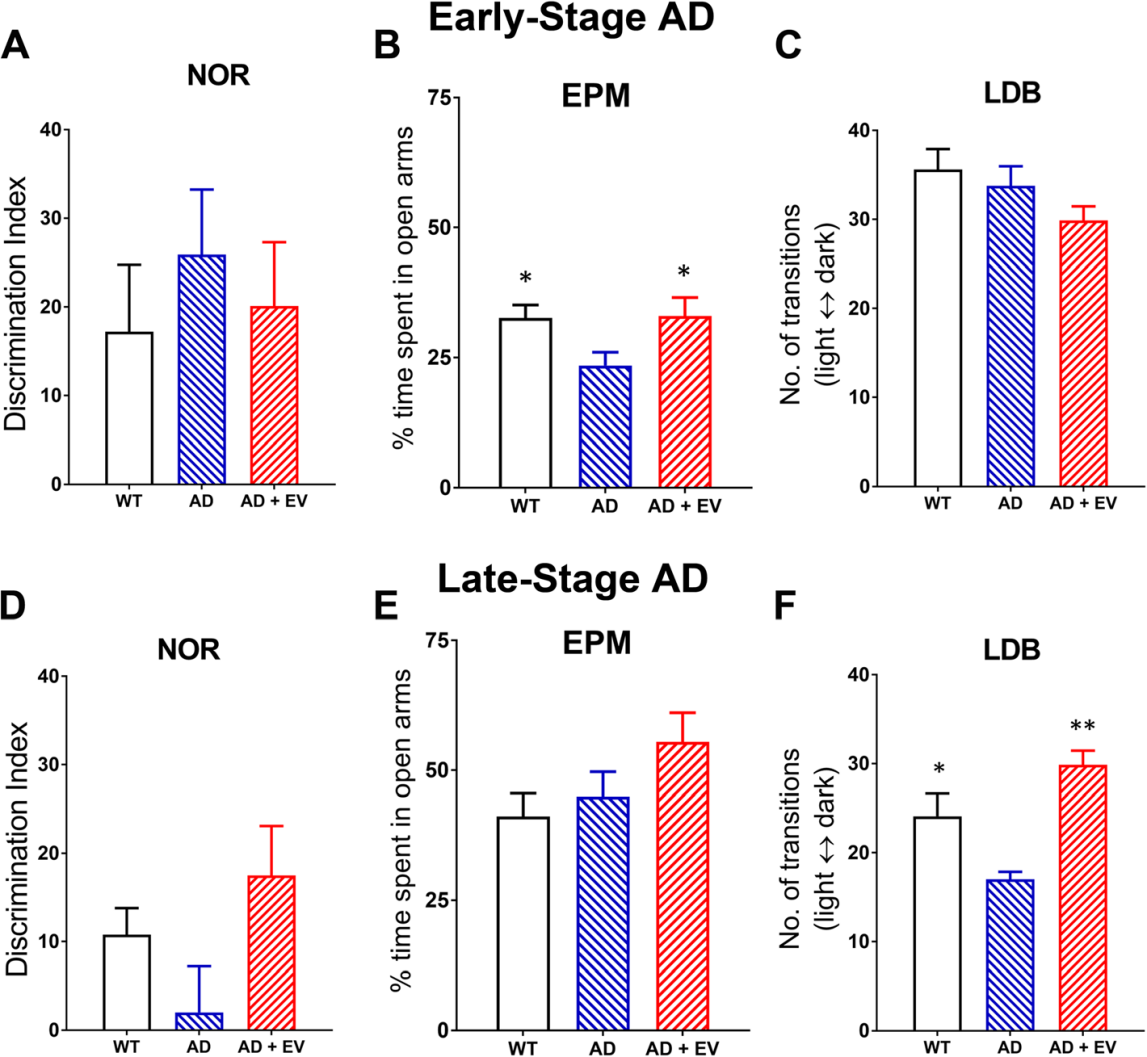
**a**–**c** Early-stage AD behavioral testing indicated no impairments on novel object recognition (NOR) or light-dark box (LDB) tests, but AD mice showed increased anxiety-like behavior on the elevated plus maze (EPM) test that was mitigated by EV treatment. **d**–**f** Late-stage AD mice exhibited no significant impairments on NOR or EPM tests, but increased anxiety-like behavior on the LDB test that was mitigated by EV treatment as shown by an increased number of transitions between the light-dark compartments. Data are presented as mean ± SEM where *N* = 14–16 mice/group. *P* values are derived from ANOVA and Bonferroni’s multiple comparisons test. *P < 0.05, **P < 0.01 as compared to AD

One month after RO injection of EVs, the mice were habituated and tested on the NOR task that engages hippocampal and medial prefrontal cortex (mPFC) function [37, 38] that may be impaired in AD. For this task, the total exploration time during the familiarization phase for each object was not different between any of the experimental groups for either the early or the late AD studies, suggesting that there were no AD-related alterations in locomotion or exploratory behavior (Supplemental Table 1). While there were no significant differences between WT, AD, and AD+EV mice during the test phase of the NOR task for either the early or late AD studies, there was a trend for decreased novel object exploration in the AD mice as compared to both WT and AD+EV cohorts of late AD mice (Fig. 3a and d, respectively).

Because altered mood frequently manifests in AD [44], we also used the EPM and LDB tests as a measure of anxiety-like behavior. Early AD male mice spent significantly less time in the open arms of the maze as compared to both WT and AD+EV mice (*P* < 0.05), suggesting increased AD-related anxiety that could be ameliorated at early disease times by a single EV treatment (Fig. 3b). However, these mice did not exhibit any reluctance to transition between light and dark compartments during LDB testing (Fig. 3c). Conversely, the late AD males exhibited no anxiety-like behavior on the EPM (Fig. 3e) but did show fewer transitions between compartments on the LDB test that were again mitigated by EV treatment (*P* < 0.01; Fig. 3f).

**Fig. 4:**
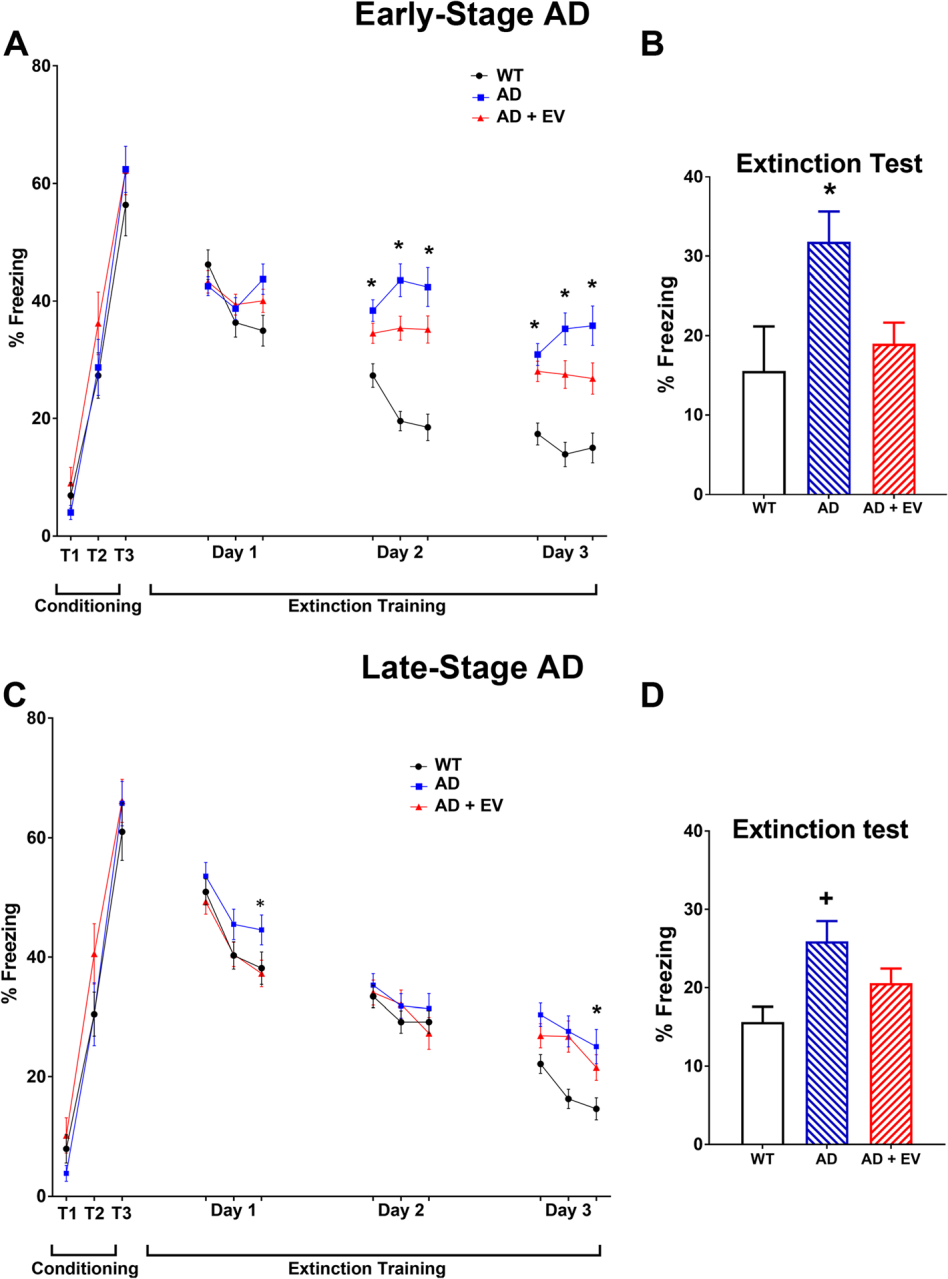
AD mice exhibited impairments in fear extinction memory and enhanced memory recall. All mice showed elevated freezing following a series of 3 tone-shock pairings (0.6 mA, T1–T3). Subsequently, fear extinction training was administered every 24 h (15 tones) for 3 days. These data are presented as the average of 5 tones (**a**, **c**). All **a** early and **c** late study mice exhibit a gradual decrease in freezing behavior (days 1–3); however, AD mice spent significantly more time freezing during the extinction training compared to controls. Twenty-four hours after extinction training, **b** early-stage AD mice exhibited enhanced fear recall (elevated freezing, *P* < 0.05), while AD mice receiving EV treatment exhibited successful extinction equivalent to that of WT control mice (reduced freezing). **d** Late-stage AD mice exhibited enhanced fear recall as compared to WT control mice. Data are presented as mean ± SEM where *N* = 14–16 mice/group. *P* values are derived from ANOVA and Bonferroni’s multiple comparisons test. *P < 0.05 as compared to WT and AD+EV, ^+^*P* < 0.05 as compared to WT.

AD mice can successfully learn the aversive association during the conditioning phase of the FE test but are impaired in dissociative learning [45]; thus, we evaluated the extinction of fear memory, the active process of memory consolidation [46]. Indeed, during the fear acquisition phase of testing, all mice from both the early and late AD cohorts showed similar percent times freezing during the 3 tone-shock pairings (Fig. 4a, c; T1–3 conditioning). During the subsequent fear extinction training in a new context, early WT and AD+EV mice demonstrated gradual decreases in freezing behavior over the day 2 and day 3 trials as compared to the AD mice that continued to freeze at a significantly higher level (Fig. 4a; day 1–3 extinction training). The results of the final day of extinction testing showed that the WT and AD+EV mice had equivalent fear extinction as illustrated by reduced percent times freezing as compared to the AD mice that continued to freeze for a significant percentage of the test time (Fig. 4b; *P* < 0.05). This AD-related impairment of fear memory extinction suggests dysfunction of neural circuitry in the hippocampus, mPFC, and amygdala [26, 45] that could be ameliorated by EV treatment. While not statistically robust, similar trends were seen in the late AD study (Fig. 4c, d).

### Stem cell-derived EVs reduce Aβ plaques in the AD brain

**Fig. 5:**
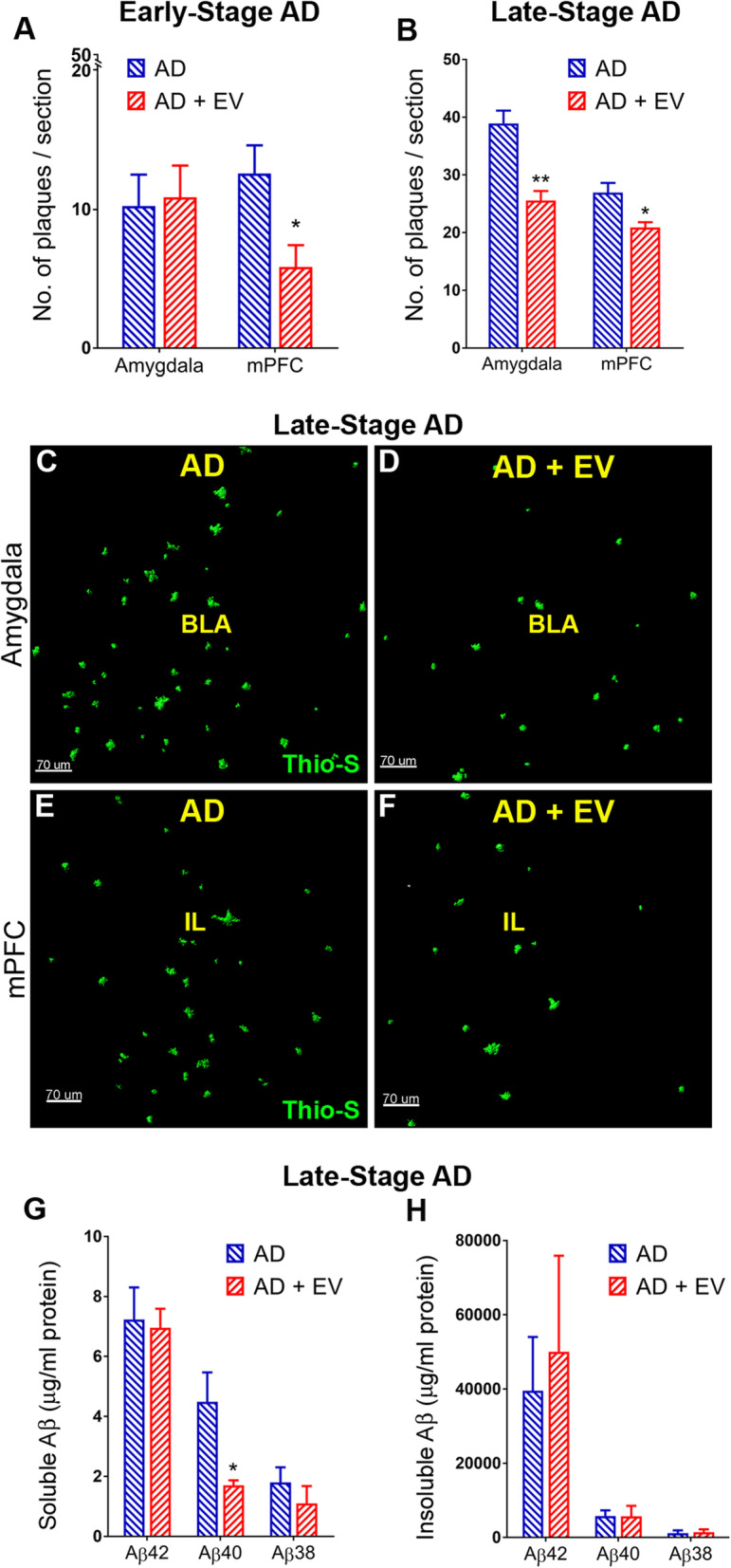
Dense core Aβ plaque number was reduced in AD mice that received EV treatment. **a** Early-stage AD mice exhibited increased numbers of Aβ plaques in the amygdala that were reduced by EV treatment and **b** late-stage AD mice exhibited increased numbers of Aβ plaques in both the amygdala and the medial prefrontal cortex (mPFC) that were reduced by EV treatment. Representative images of Thio-S staining for late-stage AD and AD+EV mice qualitatively demonstrate late AD neuropathology as shown by the accumulation of Aβ plaques in the **c** amygdala and **e** mPFC that were reduced in both the **d** amygdala and **f** mPFC regions of the AD brain by EV treatment (basal lateral amygdala, BLA; infralimbic cortex, IL; ABP, green). **g** Aβ multiplex ELISA indicated the elevation of soluble Aβ40 in brain lysates that was significantly reduced by EV treatment, **h** while no changes were observed between AD and AD+EV mice in levels of insoluble Aβ. Data are presented as mean ± SEM (Thio-S, *N* = 4 mice/group; ELISA, *N* = 7 mice/group). *P* values derived from unpaired Student’s *t* tests. *P < 0.05, **P < 0.01 as compared to AD. Scale bar = 70 μm

To determine whether EV treatment reduced amyloid pathology observed in AD, we stained for dense core plaques using Thio-S. In the early-stage AD mice that underwent EV treatment at ~ 2 months of age, the plaque load was still very low. Nonetheless, EV treatment reduced the number of plaques in the infralimbic and prelimbic areas of the mPFC (Fig. 5a; *P* < 0.05 compared to AD). In the late-stage AD mice that received EV treatment starting at 5.0–6.5 months of age, where the plaque load was significantly higher, EV treatment significantly reduced the number of Aβ plaques in both the amygdala (Fig. 5b–d; *P* < 0.01) and the mPFC (Fig. 5b, e, f; *P* < 0.005) regions of the brain. We then evaluated the levels of both soluble and insoluble Aβ_1–42_, Aβ_1–40_, and Aβ_1–38_ in the brain using triplex ELISA. While no difference between groups was observed for soluble Aβ_1–42_ and Aβ_1–38_, Aβ_1–40_ levels were significantly reduced in the brains of late-stage AD mice that had received EV treatment (Fig. 5g; *P* < 0.05). No differences were observed between AD and AD+EV mice for any species of insoluble Aβ (Fig. 5h). Together, these data suggest that just one to two EV treatments can contribute to either reduced production of Aβ or aid in clearance.

### Effect of EV treatment on activated microglia

**Fig. 6:**
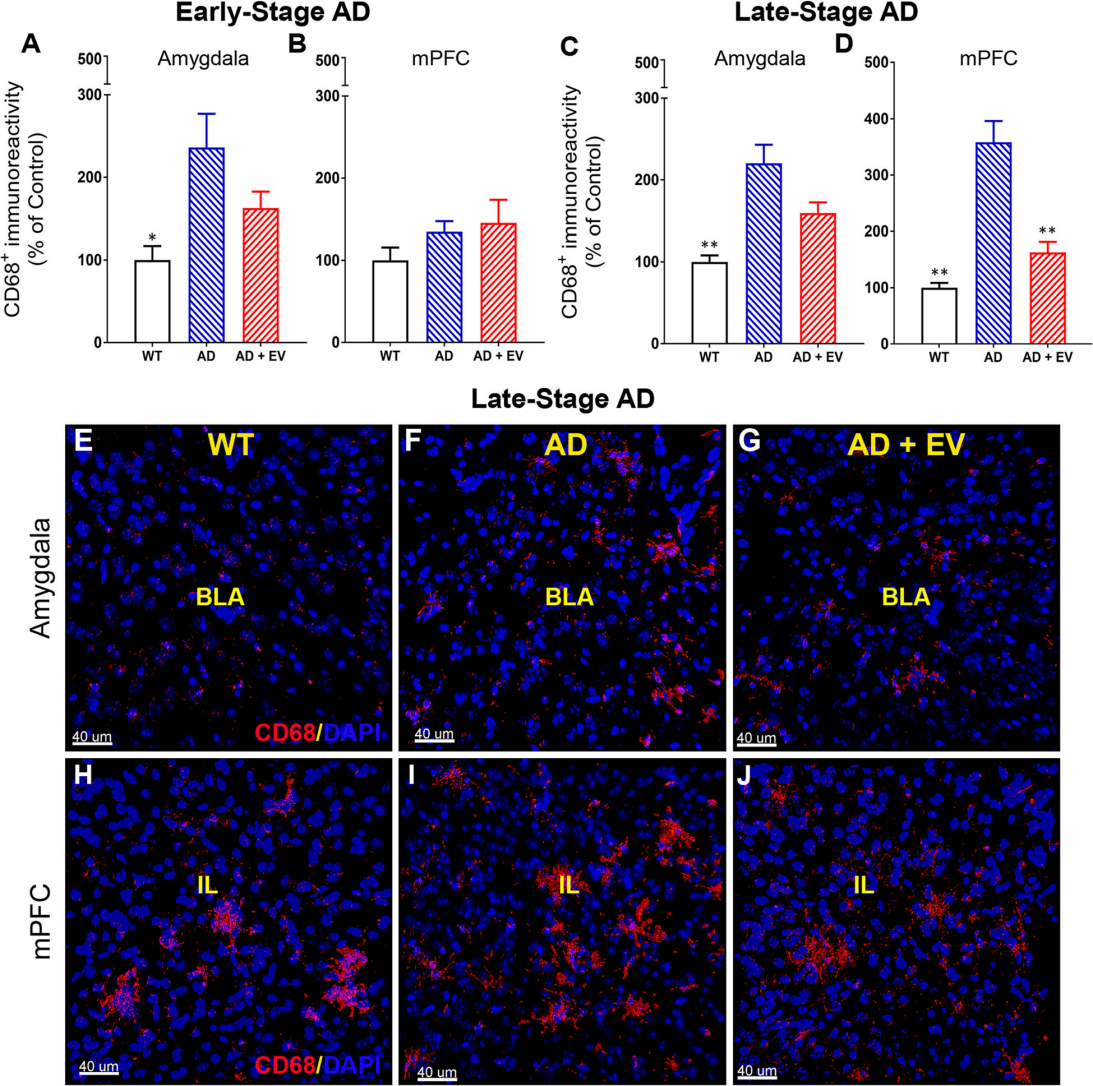
Microglial activation was reduced in AD mice that received EV treatment. Early-stage AD mice exhibited significantly increased numbers of CD68^+^ microglia in the **a** amygdala that were not significantly altered by EV treatment, and **b** no effect of disease or treatment was observed in the medial prefrontal cortex (mPFC). Late-stage AD mice showed significant increases in CD68^+^ immunoreactivity in the **c** amygdala and **d** mPFC that were ameliorated by EV treatment. Representative images of CD68 staining for late-stage mice qualitatively demonstrate these relative changes in **e**–**g** the amygdala of wild type, AD, and AD mice treated with EVs (WT, AD, AD+EV, respectively) and **h**–**j** the mPFC similarly (basal lateral amygdala, BLA; infralimbic cortex, IL; red, CD68; blue, DAPI nuclear counterstain). Data are presented as mean ± SEM (*N* = 4 mice/group). *P* values are derived from ANOVA and Bonferroni’s multiple comparisons test. *P < 0.05, **P < 0.01  as compared to AD. Scale bar = 40 μm

Microglia, the innate immune cells of the CNS, have a protective function in modulating the accumulation of Aβ plaques in the early stages of AD. However, evidence suggests that once activated, the microglia become a source of damaging inflammation and synaptic loss as the disease progresses. Using CD68 staining as a marker for microglia/macrophage activation in the brain, we observed significantly increased CD68 immunoreactivity in the amygdala of the early AD mouse brain that was not significantly reduced by EV treatment (Fig. 6a; *P* < 0.05 for AD vs WT groups); however, no significant differences among experimental groups were observed for CD68 in the mPFC region of the brain at that same time (Fig. 6b). As with the early AD mice, increased CD68 immunoreactivity was observed in the amygdala region of the brains of late AD mice, with only a trend for modulation by EV treatment (Fig. 6c, e–g; *P* < 0.01 AD vs WT groups). Unlike the early AD mice, though, late AD mice exhibit significant increases in CD68 immunoreactivity in the mPFC that were attenuated to control levels by EV treatment (Fig. 6d, h–j; *P* < 0.01 for AD vs either WT or AD+EV groups).

### EV treatment restored synaptophysin in the AD brain

**Fig. 7:**
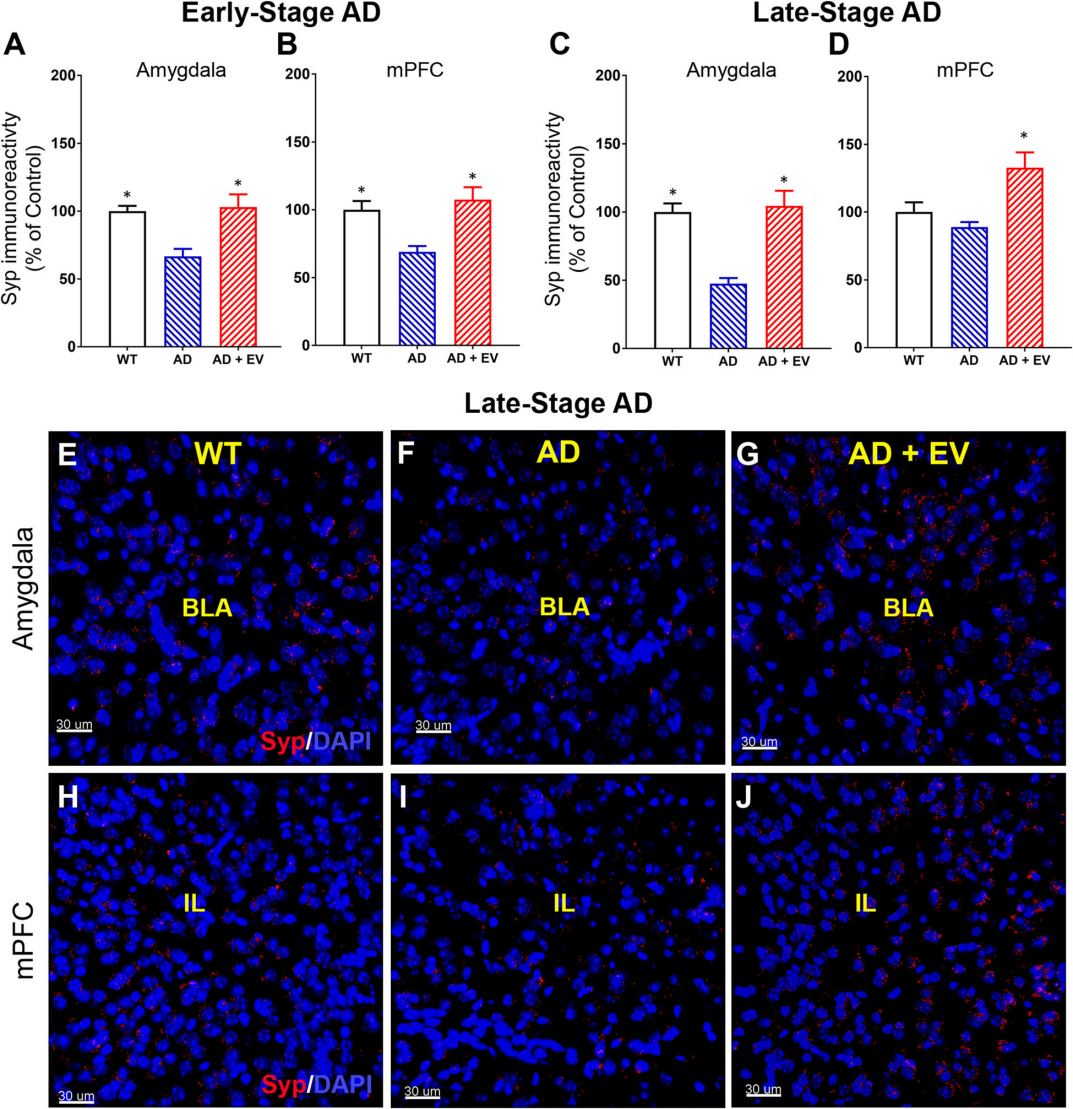
Levels of the pre-synaptic protein synaptophysin were reduced in the brains of AD mice. Evaluation of synaptophysin (Syp) immunoreactivity revealed significant decreases in the **a** amygdala and the **b** medial prefrontal cortex (mPFC) of early-stage AD mice that were ameliorated by EV treatment. Late-stage AD mice showed significant reductions in Syp in the **c** amygdala that were ameliorated by EV treatment. **d** AD-related changes in Syp were not observed in the mPFC. Representative images of Syp staining from late-stage mice qualitatively demonstrate these relative changes in **e**–**g** the amygdala of wild type, AD, and AD mice treated with EVs (WT, AD, AD+EV, respectively) and **h**–**j** the mPFC similarly (basal lateral amygdala, BLA; infralimbic cortex, IL; red, Syp; blue, DAPI nuclear counterstain). Data are presented as mean ± SEM (*N* = 4 mice/group). *P* values are derived from ANOVA and Bonferroni’s multiple comparisons test. *P < 0.05  as compared to AD. Scale bar = 40 μm

Significant loss of pre- and post-synaptic proteins, such as pre-synaptic density protein-95 and synaptophysin, respectively, has been linked to the cognitive impairments associated with AD. Evaluation of synaptophysin immunoreactivity by confocal microscopy and volumetric quantification revealed significant decreases in this pre-synaptic marker in both the amygdala and the mPFC in the early AD brain that was restored to WT control levels in the EV-treated AD brain (Fig. 7a, b; *P* < 0.05 for AD vs either WT or AD+EV groups). Similar, reductions were observed in the amygdala of the late AD brain that was also restored to WT levels by EV treatment (Fig. 7c, e–g; *P* < 0.05 for AD vs either WT or AD+EV groups). While no significant differences in synaptophysin were observed in the comparison of the mPFC region of the brain for the WT and AD groups of late AD mice, the EV treatment increased the levels of synaptophysin to greater than that of either WT or AD mice (Fig. 7d, h–j; *P* < 0.05 for AD vs AD+EV groups).

### The effect of EVs on peripheral inflammatory responses in AD

**Fig. 8:**
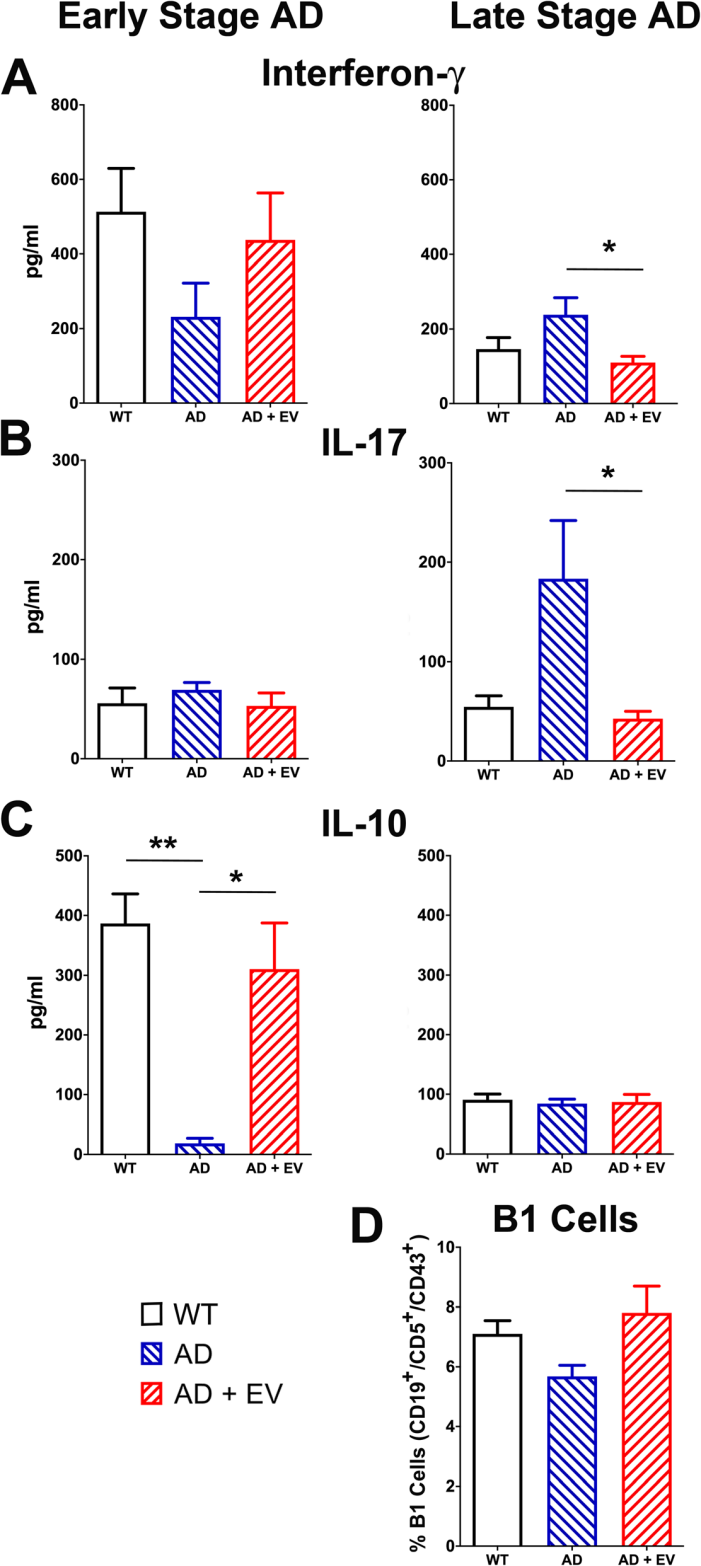
Elevated levels of inflammatory cytokines in AD mice were reduced by EV treatment. Levels of cytokines secreted by PMA- and ionomycin-stimulated spleen cells were measured in WT, AD, and AD+EV mice. **a**, **b** While unaffected in early-stage AD mice, interferon-γ and IL-17 pro-inflammatory cytokines were significantly elevated in late-stage AD mice and significantly reduced by EV treatment. **c** Alternatively, reduced levels of the anti-inflammatory cytokine IL-10 in early-stage AD mice were restored to nearly control levels in the AD mice that received EV treatment. No changes among groups were observed for IL-10 in the late-stage animals. **d** Spleen cells were also stained for B1 cells (CD19^+^, CD5^+^, CD43^+^) and analyzed by flow cytometry. While not statistically significant, a trend for a reduced percentage of B1 cells was observed for AD mice and improved by EV treatment. Data are presented as mean ± SEM (*N* = 7 mice/group). *P* values are derived from ANOVA and Bonferroni’s multiple comparisons test. *P < 0.05, **P < 0.01

The increased neuroinflammation observed in AD is frequently associated with significant peripheral immune responses that correlate with adverse outcomes in human AD patients [3]. Therefore, the levels of inflammatory and anti-inflammatory cytokines in the plasma were evaluated. Interferon-γ (IFNγ) is a pro-inflammatory cytokine that has been shown to prime microglia under pathological conditions including AD. While it remained unaffected in the early AD spleen, IFNγ was upregulated in the late-stage AD spleen, and EV treatment significantly reduced those levels (Fig. 8a; *P* < 0.05 for AD vs AD+EV groups). IL-17 overexpression has similarly been implicated in neuropathologies and AD. It was also found to be unaltered in early-stage AD, but significantly elevated in the late AD mice, and reduced WT levels by EV treatment (Fig. 8b; *P* < 0.05 for AD vs AD+EV groups). Conversely, the anti-inflammatory cytokine IL-10 that downregulates the expression of inflammatory cytokines was found to be dramatically reduced in early-stage AD mice as compared to either WT or EV+AD mice (Fig. 8c; *P* < 0.01 and *P* < 0.05, respectively for AD vs WT and AD+EV). It has been shown that the ratio of IgM to IgG is significantly decreased in human AD patients and in the 5xFAD mouse model of AD [3]. A specialized subset of B1 cells produce IgM, the percentage of which was shown to be reduced in the AD mouse. Similarly, in this study, while not significant, a trend for decreased B1 cells was observed in late-stage AD mice as compared to WT or AD+EV mice (Fig. 8d).

### Effect of EV treatment on mRNA levels in the hippocampus of the AD brain

**Fig. 9:**
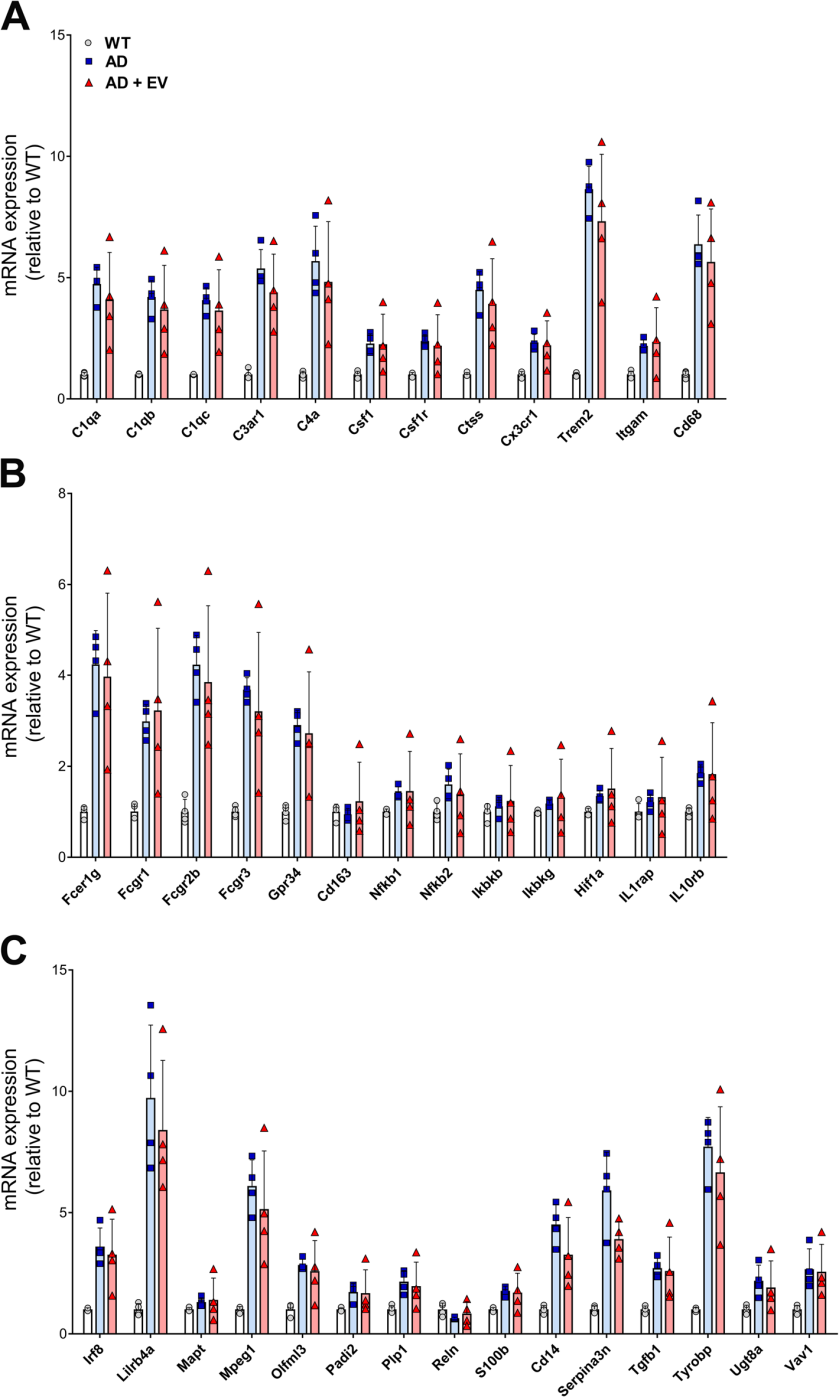
Gene expression in the hippocampus of late-stage AD mice. Analysis of **a** microglial, **b** pro-inflammation, **c** immune response, and other mRNA levels demonstrated increased gene expression in the hippocampus of late-stage AD mice as compared to WT controls. Those elevated mRNA levels were reduced in some, but not all AD+EV-treated mice. Data are presented as mean ± SEM (*N* = 4 mice/group)

Significant changes in transcription levels of genes implicated in AD pathology have been observed previously. In the current study, increases in expression for genes involved in microglial activation (Fig. 9a), pro-inflammatory signaling (Fig. 9b), and immune responses (Fig. 9c) were noted when comparing WT and late-stage AD groups of mice. Statistical comparison of AD and AD+EV mice demonstrated a subtle trend for the effectiveness of the EV treatment in certain animals; however, one AD+EV mouse remained a consistent non-responder to EV treatment, exhibiting high levels of expression for all AD-related genes evaluated.

### miRNA microarray analysis reveals potential therapeutic EV cargo

To investigate potential functional components of hNSC-derived EVs, miRNA cargo was evaluated using a targeted human miRNA array [11] (Supplemental Table 2). Candidate miRNA implicated in AD-related pathways including learning, memory, and neuroinflammation were identified by cross-referencing the array data with the literature, which vary widely in opinions regarding the pathways influenced by a particular miRNA and whether the effect is beneficial or detrimental to CNS homeostasis or pathology. Among the miRNA identified in this analysis were miR-125b-5p, miR-124-3p, and miR-125a-5p (Table 1). All of these select candidates were confirmed to be present in EV RNA samples using TaqMan Advanced miRNA Assays, providing candidate miRNA for potential follow-up studies that could influence CNS function [55–57].

**Table 1:** Candidate microRNA EV cargo

MicroRNA	Average miR expression (log scale)	Relevance to AD	Reference
**MiR-124-3p**	**20,761.5**	**Inhibition of neural inflammation in the damaged brain** **Downregulated in AD and neurodegenerative diseases**	[20, 47–51]
MiR-125b-5p	22,278.9	Normal expression attenuates Aβ-induced toxicity, reduces apoptosis and oxidative stress Regulates PSD-95 levels	[52, 53]
MiR-125a-5p	5284.8	Downregulation decreases levels of PSD-95 in dendrites and dendrite complexity	[54]
Neg control 1	726.0		
Neg control 2	709.0		
Neg control 3	688.5		

## Discussion

AD is an irreversible neurodegenerative disease for which there are currently no effective therapies to slow or reverse the disease progression. A typical hallmark of AD is the accumulation of Aβ plaques that leads to persistent microglial activation creating a pro-inflammatory, neurotoxic environment that ultimately causes cognitive and functional decline and memory loss. Our past work has defined the effects of clinically relevant radiation exposures on the brain, which include cognitive impairment, neuroinflammation, dysregulation of pre- or post-synaptic protein levels, and loss of neuronal structure [5, 6]. Work then demonstrated that cranially grafted hNSC and then hNSC-derived EVs were effective in mitigating radiation-induced neurodegenerative events. Here, we have demonstrated that the same hNSC-derived EV therapy is effective in ameliorating AD-related neurodegenerative pathologies. Single or duplicate EV treatments were found to reverse cognitive impairments in AD mice, including anxiety-like behaviors and fear memory consolidation.

One of the hallmarks of AD pathology is a progressive accumulation of Aβ plaques in the brain. While plaque density is not directly linked with dementia [58, 59], the neurodegenerative effects of plaques in triggering neuroinflammation and loss of neurons are well documented [4, 60, 61]. To delineate the regenerative effects of stem cell-derived EVs on pathology, whole-brain ELISA on late-stage AD brains was employed and revealed significant reductions in the levels of soluble amyloid-β_1–40_, that along with Aβ_1–42_, is a robust predictor of AD-related synaptic loss [62–64]. While it is unclear why no changes in levels of Aβ_1–42_, our Thio-S staining for dense core Aβ plaques confirmed that EV treatment provided some reductions in plaque load. These data indicate that the neuroprotective effects of EVs are exerted, in part, by reducing the plaque burden in the AD brain at early and late stages of disease progression.

Persistent activation of microglia in the AD brain is detrimental to neuronal and cognitive function. Our past data have shown anti-inflammatory effects of stem cells or stem cell-derived EVs in clinically relevant irradiation [8, 9, 65] and chemobrain [66] models. Substantiating the neuroprotective impact of EVs in the AD brain, we found significant reductions in the levels of CD68, a marker of microglial/myeloid cell activation. While we did not evaluate neuron structure in the current study, persistent microglial reactivity has been linked to synapse loss in AD [61, 67, 68]. Mouse models of AD, including the 5xFAD model, have also been found to have reduced levels of pre- and post-synaptic protein levels. Our data demonstrated significant reductions in synaptophysin immunoreactivity in the AD brain that were restored by EV treatment. Together, these data in an AD model, as well as our studies of EV amelioration of radiation-induced brain injury, suggest dysfunction of neural circuitry in the hippocampus, mPFC, and amygdala that could be ameliorated by EV treatment [10–12].

Inflammatory markers from the peripheral immune response indicate a highly pro-inflammatory environment in AD mice that could be mitigated in part by EV treatment. IFNγ and IL-17 are highly inflammatory cytokines, and both implicated in CNS-related disorders such as multiple sclerosis. Reduction in IL-17 is especially important as IL-17 has been shown to cause neuronal cell death in the human Parkinson’s disease iPSC model [69]. Decreased levels of IL-10 levels, an anti-inflammatory cytokine in early AD, also indicate an impairment in controlling inflammation. Reductions in B1 cells in late-stage AD may also contribute to AD-related accumulation of Aβ plaques as these cells contribute significantly to the removal of cell debris and self-proteins. Restoration of B1 cell frequencies and reduced IFNγ and IL-17 by EVs at late-stage AD combined with increased IL-10 levels at the early stage indicates that EV treatment is affecting multiple pathways to dampen inflammation.

The cognitive and molecular observations from this study are supported by gene expression analysis from the late-stage AD brain that demonstrated consistent increases in mRNA levels for genes involved in microglial activation, inflammation, and other AD pathologies. Critically, the two EV treatments to the late-stage AD mouse resulted in strong trends for reductions in that gene overexpression as compared to AD only mice.

An important part of this study is the analysis and identification of potentially beneficial EV cargo. Array analysis identified at least 3 strong candidate miRNA, miR-125a, miR-125b, and miR-124. While overexpression of any of these candidate miRNA can be damaging in specific cases [47], literature has also suggested they serve beneficial roles in the CNS. MiR-125a and miR-125b have been shown to maintain PSD-95 levels in dendrites and regulate synaptic structure, and miR-125b is an important regulator of synaptic structure and function [52, 54]. MiR-124 was the clear lead candidate EV miRNA in the context of ameliorating the hallmarks of AD. MiR-124 expression has been demonstrated to reduce neuroinflammation and promote neurite outgrowth in traumatic brain injury models [20, 48–50]. Further, miR-124 has been suggested to regulate glycogen synthase kinase 3β and glucocorticoid receptor levels in the context of AD (GSK3β and GR, respectively) and regulate anxiety as well as impulse control disorders that are indicative of poor decision-making in humans [51]. The reported downregulation of miR-124 in the AD brain may, at least in part, contribute to the neuropathological phenotypes associated with AD. Significantly, we have recently demonstrated that AAV9-mediated overexpression of miR-124 alone was able to reduce the neurodegenerative consequences of cranial irradiation on cognition and microglial activation [11]. We hypothesize that similar pathways may be at play in the AD brain, where miR-124 helps to resolve a wide range of neuropathologies.

Similar observations regarding the efficacy of EVs in the treatment of AD have been made by other laboratories as well. Li and colleagues used embryonic mouse NSC-derived EVs to treat 9-month-old B6C3-tg mice via stereotactic injection into the lateral ventricles [14]. Five weeks later, AD mice treated with EVs were shown to have improved performance on the Morris water maze as compared to untreated AD littermates. Similarly, the EV treatment increased the levels of synaptic proteins, including synaptophysin and PSD-95, and improved synaptic morphology and mitochondrial function. While the EV treatment also reduced the levels of pro-inflammatory cytokines, it did not reduce the Aβ plaque load, supporting the assertion that plaques might not drive the cognitive phenotype of AD. Using mouse mesenchymal stem cell-derived EVs, Losurdo and colleagues administered two intranasal doses to 3xTg AD mice and found a shift towards an anti-inflammatory phenotype and reduced activation of microglia, as well as improved dendritic spine density [15]. These examples using two distinct models of AD corroborate our data on the beneficial effects of systemic injections of EVs on disease pathology.

### Limitations

While we do not see complete remediation of cognitive function, one or two injections of EVs were able to partially restore cognitive indices in male 5xFAD mice. Our current observations suggest that additional, ongoing treatments with the EVs could improve the efficacy of the therapy, forestalling or even reversing AD pathologies. Additional studies are needed to confirm the efficacy of this same EV therapeutic strategy in the treatment AD in female mice. Importantly, we emphasize that our studies provide indications of at least one of the mechanisms by which these hNSC-derived EVs may repair the AD brain, delivering miRNA capable of reducing inflammation and protecting neuronal structure, possibly through epigenetic regulation of gene expression. We acknowledge that other cargo is likely to play a role in the effectiveness of these EVs and future studies will focus not only on the miRNA cargo and mechanisms but also on the other potentially beneficial EV cargo such as protein.

## Conclusion

AD is an irreversible neurodegenerative disease affecting millions of people worldwide, and regenerative therapies to mitigate AD neuropathology have shown very limited success. The findings of this study demonstrate the neuroprotective efficacy of systemic administration of stem cell-derived EVs for remediation of behavioral and molecular AD neuropathologies. Further, these data suggest that EV-contained miRNA may represent a potential, specific mechanism for follow-up studies to develop therapeutic strategies to meet this critical unmet medical need.

## Supplementary Information

**Additional file 1.**

## Data Availability

Correspondence and request for data or materials should be addressed to JEB.

## Abbreviations

Aβ: Amyloid-beta
AD: Alzheimer’s disease
BLA: Basal lateral amygdala
CNS: Central nervous system
ELISA: Enzyme-linked immunosorbent assay
EPM: Elevated plus maze
EV: Extracellular vesicle
FE: Fear extinction
HSCRO: Human Stem Cell Research Oversight Committee
hNSC: Human neural stem cell
IL: Infralimbic cortex
IFNγ: Interferon gamma
IL: Interleukin
LDB: Light-dark box
MiR, MiRNA: MicroRNA
mPFC: Medial prefrontal cortex
NIH: National Institute of Health
NOR: Novel object recognition
PFA: Paraformaldehyde
PSD-95: Post-synaptic density protein-95
RO: Retro-orbital
Syp: Synaptophysin
Thio-S: Thioflavin-s
WT: Wild type
