# Supplementary material for: Human neural stem cell-derived extracellular vesicles mitigate hallmarks of Alzheimer’s disease
Source: Alzheimers Res Ther. 2021 Mar 6;13:57. doi: 10.1186/s13195-021-00791-x (PMC7937214; doi:10.1186/s13195-021-00791-x)
Supplement: Supplementary file 1 — Additional file 1. [file 13195_2021_791_MOESM1_ESM.docx]

**Supplemental Information**

Apodaca *et al.*

**Supplemental Data**

**Supplemental Table 1**

| **NOR Early-Stage AD** | **Time spent exploring novel object**  **(sec)** | **Time spent exploring familiar object**  **(sec)** | **Total exploration time**  **(sec)** |
| --- | --- | --- | --- |
| WT | 14.07 ± 1.903 | 9.21 ± 0.911 | 23.28 ± 2.252 |
| AD | 16.64 ± 2.254 | 9.59 ± 1.449 | 26.22 ± 2.779 |
| AD + EV | 15.02 ± 1.838 | 10.12 ± 1.119 | 25.14 ± 2.633 |

| **NOR Late-Stage AD** | **Time spent exploring novel object**  **(sec)** | **Time spent exploring familiar object**  **(sec)** | **Total exploration time**  **(sec)** |
| --- | --- | --- | --- |
| WT | 19.36 ± 2.609 | 13.12 ± 1.890 | 34.09 ± 4.522 |
| AD | 18.65 ± 1.421 | 18.85 ± 1.962 | 37.51 ± 2.791 |
| AD + EV | 17.36 ± 1.634 | 12.39 ± 1.569 | 29.75 ± 2.989 |

Total exploration time for the novel object recognition (NOR) task does not vary significantly between the groups. Novel object, familiar object, and total exploration times are presented for the NOR task for the early-stage and late-stage AD cohorts. Data are presented as mean + S.E.M. (N=14-16 mice/group). See Methods for groups and behavioral testing protocol details. (WT, wild type; AD Alzheimer’s disease; EV, extracellular vesicle)

**Supplemental Table 2. List of microRNAs detected in hNSC-derived EV by miRNA microarray.**

| Gene ID | Name | Expression |
| --- | --- | --- |
| 169399 | hsa-miR-4750-5p | 65275.40 |
| 148687 | hsa-miR-1908-5p | 65269.30 |
| 169272 | hsa-miR-4419b | 65269.00 |
| 169285 | hsa-miR-4467 | 65264.40 |
| 168802 | hsa-miR-4516 | 65257.00 |
| 169082 | hsa-miR-1275 | 65254.00 |
| 169024 | hsa-miR-3960 | 65251.90 |
| 168619 | hsa-miR-1260b | 65249.50 |
| 147604 | hsa-miR-4285 | 65243.80 |
| 169375 | hsa-miR-660-3p | 65243.30 |
| 169050 | hsa-miR-4787-5p | 65242.60 |
| 147767 | hsa-miR-4279 | 65240.80 |
| 168978 | hsa-miR-371b-5p | 65239.80 |
| 169034 | hsa-miR-642b-5p | 65237.60 |
| 148156 | hsa-miR-3686 | 65235.10 |
| 169380 | hsa-miR-3124-3p | 65233.00 |
| 169313 | hsa-miR-4800-3p | 65226.00 |
| 169282 | hsa-miR-4290 | 65224.60 |
| 168878 | hsa-miR-5100 | 65197.80 |
| 169015 | hsa-miR-4454 | 65196.10 |
| 148682 | hsa-miR-483-3p | 65164.80 |
| 169028 | hsa-miR-4708-3p | 65111.10 |
| 168637 | hsa-miR-3940-5p | 65069.40 |
| 169188 | hsa-miR-4443 | 65047.80 |
| 168638 | hsa-miR-4530 | 64956.50 |
| 147942 | hsa-miR-4268 | 64800.50 |
| 146072 | hsa-miR-1469 | 57462.40 |
| 168870 | hsa-miR-1246 | 49278.60 |
| 168925 | hsa-miR-1273g-3p | 45274.60 |
| 169316 | hsa-miR-3976 | 40212.00 |
| 169395 | hsa-miR-4484 | 35823.80 |
| 169393 | hsa-miR-4747-5p | 35632.40 |
| 147701 | hsa-miR-491-3p | 34284.00 |
| 169169 | hsa-miR-5684 | 32572.10 |
| 169130 | hsa-miR-4764-3p | 28517.00 |
| 147667 | hsa-miR-3182 | 27494.10 |
| 169381 | hsa-miR-4421 | 27359.10 |
| 168844 | hsa-miR-4532 | 26934.40 |
| 10138 | hsa-miR-130a-3p | 26912.80 |
| 169183 | hsa-miR-4644 | 26901.10 |
| 147722 | hsa-miR-4306 | 26528.80 |
| 168998 | hsa-miR-4508 | 26315.80 |
| 169031 | hsa-miR-4726-5p | 25628.10 |
| 169110 | hsa-miR-4497 | 25622.30 |
| 30787 | hsa-miR-125b-5p | 22278.90 |
| 168893 | hsa-miR-4505 | 21654.80 |
| 42581 | hsa-miR-513a-5p | 20892.80 |
| 14328 | hsa-miR-124-3p | 20761.50 |
| 168882 | hsa-miR-664b-3p | 20614.60 |
| 168944 | hsa-miR-4707-5p | 20297.30 |
| 168798 | hsa-miR-4668-5p | 19433.50 |
| 145768 | hsa-miR-665 | 19415.10 |
| 168640 | hsa-miR-4475 | 19240.60 |
| 10967 | hsa-miR-16-5p | 18849.10 |
| 147817 | hsa-miR-3196 | 18224.30 |
| 42832 | hsa-miR-638 | 18219.10 |
| 27568 | hsa-miR-744-5p | 17290.60 |
| 169336 | hsa-miR-17-5p | 16147.60 |
| 168568 | hsa-miR-1290 | 15757.40 |
| 169087 | hsa-miR-149-3p | 15612.60 |
| 168661 | hsa-miR-4531 | 15201.80 |
| 169143 | hsa-miR-4459 | 14708.00 |
| 42923 | hsa-miR-30c-5p | 14505.10 |
| 148317 | hsa-miR-3621 | 14323.40 |
| 145845 | hsa-miR-20a-5p | 14280.80 |
| 148032 | hsa-miR-3685 | 13802.00 |
| 168868 | hsa-miR-5681b | 13785.80 |
| 148493 | hsa-miR-3613-3p | 13544.40 |
| 169407 | hsa-miR-4301 | 13448.90 |
| 145846 | hsa-let-7e-5p | 13301.50 |
| 30687 | hsa-miR-93-5p | 13220.10 |
| 19582 | hsa-miR-106b-5p | 13131.00 |
| 169244 | hsa-miR-5572 | 12811.80 |
| 42696 | hsa-miR-943 | 12697.30 |
| 42761 | hsa-miR-675-5p | 12573.80 |
| 168919 | hsa-miR-4456 | 12266.00 |
| 42442 | hsa-miR-498 | 12264.60 |
| 42502 | hsa-miR-204-3p | 12235.10 |
| 148481 | hsa-miR-3646 | 12227.00 |
| 169009 | hsa-miR-548ap-5p/hsa-miR-548j-5p | 12156.90 |
| 42654 | hsa-miR-483-5p | 11897.00 |
| 147506 | hsa-miR-21-5p | 11557.60 |
| 169239 | hsa-miR-4732-5p | 11333.10 |
| 169182 | hsa-miR-4728-3p | 10642.40 |
| 10936 | hsa-miR-130b-3p | 10083.30 |
| 147588 | hsa-miR-4288 | 9881.38 |
| 146158 | hsa-miR-3202 | 9815.25 |
| 169129 | hsa-miR-4284 | 9098.88 |
| 13143 | hsa-miR-301a-3p | 9095.75 |
| 169204 | hsa-miR-4709-3p | 8983.13 |
| 27565 | hsa-miR-423-5p | 8745.75 |
| 169102 | hsa-miR-4639-3p | 8722.13 |
| 169295 | hsa-miR-4725-3p | 8510.25 |
| 46801 | hsa-miR-106a-5p | 8501.38 |
| 145693 | hsa-miR-92a-3p | 8388.75 |
| 10919 | hsa-miR-103a-3p | 8305.88 |
| 169070 | hsa-miR-4695-3p | 7865.75 |
| 168672 | hsa-miR-1587 | 7575.88 |
| 169412 | hsa-miR-1260a | 7287.25 |
| 11023 | hsa-miR-222-3p | 7136.63 |
| 11245 | hsa-miR-433-5p | 6929.88 |
| 147614 | hsa-miR-4299 | 6853.38 |
| 42509 | hsa-miR-219a-5p | 6802.88 |
| 169305 | hsa-miR-4455 | 6785.00 |
| 146196 | hsa-miR-711 | 6645.50 |
| 46731 | hsa-miR-4657 | 6630.38 |
| 168653 | hsa-miR-3158-5p | 6549.25 |
| 4040 | hsa-miR-9-5p | 6548.75 |
| 148228 | hsa-miR-3656 | 6466.75 |
| 169322 | hsa-miR-4534 | 6427.75 |
| 148465 | hsa-miR-3611 | 6334.75 |
| 168815 | hsa-miR-4488 | 6281.00 |
| 169170 | hsa-miR-4472 | 6255.00 |
| 169409 | hsa-miR-4286 | 6149.88 |
| 42640 | hsa-miR-20b-5p | 5880.00 |
| 148622 | hsa-miR-877-3p | 5796.75 |
| 169079 | hsa-miR-4667-5p | 5611.50 |
| 29852 | hsa-miR-9-3p | 5573.50 |
| 11077 | hsa-miR-363-3p | 5559.50 |
| 169023 | hsa-miR-4712-3p | 5539.38 |
| 42839 | hsa-miR-135a-5p | 5445.75 |
| 17506 | hsa-miR-24-3p | 5336.38 |
| 17280 | hsa-miR-15b-5p | 5297.63 |
| 10928 | hsa-miR-125a-5p | 5284.75 |
| 14258 | hsa_negative_control_1 | 726.00 |

Displayed are the ID numbers, names of miRNA, and the average expression value (mean of replicate arrays). This is the complete list of miRNAs with expression values greater than or equal to miR-125a-5p, the lowest-expressed miRNA that was experimentally confirmed. The first negative control probe is shown as well for reference. (miRNA, miR, microRNA; hNSC, human neural stem cell; EV, extracellular vesicle)

**Supplemental Methods**

**Behavioral testing**

NOR testing occurred in a dimly lit test arena with a layer of fresh corncob bedding (30🞨30🞨30 cm box, 48 lux). The same bedding for each group of mice was used for the next 3 days of habituation and any object used in the arena was cleaned with 10% ethanol in between each trial. Mice were habituated to the arena with only bedding for the first three days with a single upright inverted 50 ml conical tube located directly in the middle of the arena (10 minutes /day). On the following testing day, two plastic objects that were similar in color, shape, and size were magnetically affixed 16 cm apart in the arena and each mouse was allowed 5 minutes to explore the objects. Each mouse was returned to the home cage for 5 minutes while one familiar object was substituted for a novel object, both of which had been cleaned with 10% ethanol during this 5-minute break. The mouse was then returned to the arena for 5 minutes of further exploration. Video of the test was filmed from above and manually scored for time spent interacting with familiar versus novel objects (nose within 2 cm radius). The discrimination index was then calculated for each mouse from these values: [(novel/total exploration time) – (familiar/total exploration time)] 🞨 100.

The EPM and LDB tests are based on the tendency of anxious rodents to avoid open or brightly lit areas and to exhibit reluctance to explore open environments, resulting in reduced amounts of time spent in the open arms of the EPM or reduced numbers of transitions between the dark and light compartments of the LDB testing arena [39]. The EPM consists of 2 open arms and 2 closed arms arranged such that the 2 open arms are opposite each other and at 90 degrees to the closed arms in the shape of a plus sign, elevated 40 cm off the floor, placed in a brightly lit (915 lux) room [40, 41]. Each mouse was placed in the neutral center zone of the plus maze and allowed to explore for 5 minutes. Anxiety-like behavior was scored as the percent time spent in the open arms of the maze as compared to the closed arms. Following EPM, the next day mice were tested on LDB where anxiety was measured by a mouse’s willingness to transition freely between a large well-lit chamber (30🞨20🞨27 cm, 915 lux) through a 7.5🞨7.5 cm opening to a smaller, dimly lit chamber (15🞨10🞨27 cm, 4 lux). Fewer transitions and less time spent in the well-lit chamber during the 10-minute test suggested increased anxiety-like behavior [39]. For both tasks, videos of the tests were filmed from above and manually scored for time spent in the open arms of the EPM or number of transitions in the LDB task.

The FE test relies on two distinct contexts (A and B) to determine whether mice could learn and then extinguish conditioned fear responses over 5 days [42, 43]. The conditioning testing chamber (context A; 17.5🞨17.5🞨18 cm; Coulbourn Instruments) had a steel grid floor and the scent of 0.2% acetic acid in distilled water, while the extinction chamber (context B) had a smooth Plexiglas floor, additional stimulus lighting and the scent of 10% almond extract in distilled water. Each test chamber was disinfected in between testing trials. Digital cameras were mounted in the ceiling of each chamber and connected via a quad processor for automated scoring of freezing (FreezeFrame, Coulbourn Instruments). For each mouse, the fear conditioning protocol for day 1 used context A and started with a 120 second pre-fear conditioning habituation followed by 3 pairings of a 120-second, 80dB, 16 kHz white noise conditioned stimulus (CS) co-terminating with a 1 second, 0.6 mA foot shock (US) presented at 2-minute intervals (day 1, T_1_-T_3_). For extinction training, starting on day 2, each mouse was placed in context B and allowed to acclimate for 2 minutes followed by extinction training that was comprised of 15 non-reinforced 120 second CS presentations at 5-second intervals. Fear extinction data are presented as the average of 5 tones. Extinction training was repeated daily for 2 additional days. Subsequently, retention testing was performed on day 5 where each mouse was returned to context B and following a 2 minute acclimation period freezing was assessed during three non-US reinforced CS tones (16 kHz, 80 dB, lasting 120 seconds) at 2-minute intervals. Extinction memory was calculated as the percentage of time spent freezing during the test.
